# Surface modification of cellulose nanocrystals with different acid anhydrides for improved dispersion in poly(butylene succinate)

**DOI:** 10.1039/c8ra07597b

**Published:** 2018-11-14

**Authors:** Yingying He, Jiang Zhu, Wentao Wang, Haitao Ni

**Affiliations:** College of Materials and Chemical Engineering, Chongqing University of Arts and Sciences Chongqing 402160 People's Republic of China jiangzhu415@163.com; Chongqing Key Laboratory of Environmental Materials & Remediation Technology, Chongqing University of Arts and Science Chongqing 402160 People's Republic of China htniok@163.com

## Abstract

Hydrophilic cellulose nanocrystals (CNC) are typically poorly dispersed in non-polar polymer matrices and, hence, a method for the surface modification of CNC is developed for improving this dispersion. This method included an esterification reaction with acetic anhydride, butyric anhydride, and caproic anhydride. The particle size distribution (range of sizes: 80–310 nm) of CNC was determined. The SEM-EDAX indicated that (i) the structure of CNC was maintained even after incorporation of the acid anhydride and (ii) the carbon C content of modified-CNC was higher than that of pure CNC. The chemical structure of modified-CNC was identified *via* FT-IR and ^13^C NMR spectroscopy. The contact angle of CNC and modified-CNC with water and methylene iodide was measured. The surface energy of modified-CNC was lower than that of pure CNC. Thermal-property measurements indicated that the initial decomposition temperature (based on 5 wt%) of the modified-CNC was slightly higher than that of CNC. Poly(butylene succinate) (PBS) composites were obtained by mixing modified-CNC into a PBS matrix *via* simple melt blending of a double screw. The PBS/modified-CNC composites were investigated *via* DSC and XRD. Tensile testing indicated that the tensile modulus improved gradually with increasing modified-CNC content, whereas the elongation at fracture decreased.

## Abstract 2

The hydrophobic CNC was dispersed in PBS to become a high dispersity and mechanical property and thermal stability PBS/modified-CNC composite.

## Graphical abstract

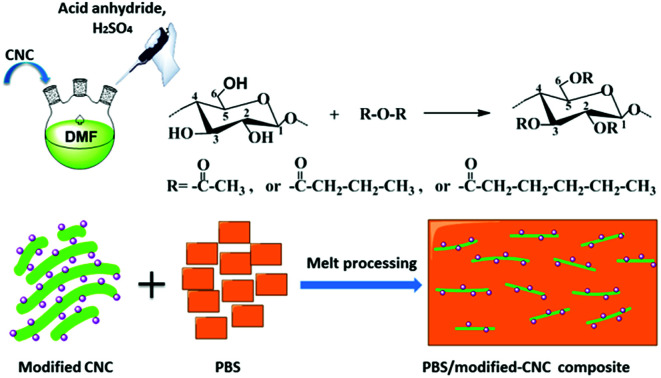

## Introduction

Owing to severe environmental pollution, renewable and biodegradable resources have become increasingly important for industry.^1^ Easily accessible materials with excellent biodegradability are urgently needed. Cellulose, which is one of the most ample biopolymers on Earth, can be obtained from various sources including wood, cotton, hemp, and other plant-based materials.^2–4^ Cellulose nanocrystals (CNC) have attracted significant interest, owing to intrinsic properties, such as nanoscale dimensions, remarkable mechanical properties, large surface area, excellent flexural rigidity, and biodegradability. In addition, intermolecular and intramolecular hydrogen bonding occurs between hydroxyl groups of the main chain comprising CNC.^5,6^ CNC is an ideal reinforcing agent for biodegradable polyesters including poly(lactic acid) (PLA), polyvinyl acetate (PVA), and poly(butylene succinate) (PBS).^7–9^ CNC and cellulose derivatives, which are applicable to various fields, are considered workable alternatives to non-biodegradable materials.^10,11^

Several properties of polymer composites depend significantly on the dispersion state of nanoparticles, *i.e.*, the reinforcing effects result from the percolating network of CNC and excellent interfacial compatibility between the matrix and the fillers.^12^ Nevertheless, the hydrogen bonding between the three hydroxyl groups comprising the repeat unit of CNC occurs in aggregates. Furthermore, owing to differences in the corresponding polarities, CNC is poorly dispersed in non-polar polymer matrices. Methods for CNC modification, such as esterification, graft copolymers, acetylation, and silylation, have been developed with the aim of overcoming this problem.^13–16^ The dispersion of CNC may be improved, for example, by reducing the number of hydroxyl groups *via* esterification.

PBS is biodegradable synthetic polyester that exhibits good biocompatibility, and has the potential for widespread use in various industrial applications (such as garbage bags, packaging bags, and agricultural materials). However, PBS suffers from drawbacks such as low rigidity, high cost, and a low rate of degradation.^17–19^ To overcome these drawbacks, and expand the range of PBS applications, PBS composites enhanced with natural reinforcements (such as coffee shell-fiber, straw fiber, and bamboo fiber) have been proposed.^20–24^

According to the aforementioned studies, dispersion and interfacial adhesion of polymer composites are very important factors and, hence, improving the dispersion of CNC nanoparticles in non-polar PBS polymer matrices is essential. In the present study, CNC was obtained *via* the acid hydrolysis of micro-crystalline cellulose (MCC). Accordingly, CNC was modified with different length chains of acid anhydrides. Through an esterification reaction, hydrogen in the hydroxyl group of CNC was substituted by a hydrophobic ester group. The modified-CNC would promote interfacial adhesion with PBS. The chemical structures of CNC and modified-CNC were identified *via* Fourier transform infrared (FT-IR) and ^13^C nuclear magnetic resonance (NMR) spectroscopy. Thermogravimetric analysis (TGA) was performed on CNC and modified-CNC nanoparticles and the contact angles with water and methylene iodide were measured in each case. Similarly, the surface free energy of CNC and modified-CNC was calculated. The corresponding PBS/modified-CNC composites were prepared *via* simple melt blending of a double screw, and the morphology as well as the crystalline structure of these composites was investigated. The reinforcing effect of modified-CNC was investigated *via* differential scanning calorimetry and mechanical-property characterization.

## Experimental

### Materials

Microcrystalline cellulose (MCC, column chromatography) was obtained from Sinopharm Chemical Reagent Co., Ltd (China). Butyric anhydride (b, AR) and caproic anhydride (c, AR) were supplied by Aladdin. Sulfuric acid (H_2_SO_4_, AR) at a purity level of 98.08%, *N*,*N*-dimethylformamide (DMF, AR), acetic anhydride (a, AR), and acetone (AR) were all produced by Chongqing Chuandong Chemical (group) Co., Ltd (China). Methylene iodide (CH_2_I_2_, AR), and potassium bromide (KBr, GR) were provided by the Fuyu Fine Chemical Institute of Tianjin. Commercial poly(butylene succinate) (PBS) particles with a relative density of 1.26 and grade equivalent to that of an extrusion sheet were purchased from Anqing Hexing Chemical Co., Ltd. (China).

### Preparation of modified cellulose nanocrystals

CNC was obtained *via* H_2_SO_4_ hydrolysis of MCC. The particle size distribution (range of sizes: 80–310 nm) of CNC was measured *via* dynamic light scattering (DLS; NanoBrook 90 Plus Zeta, Bruker).^25,26^ A portion (*i.e.*, 31.25 g) of MCC was mixed with 64 wt% H_2_SO_4_ aqueous solution (500 mL). The mixture was then vigorously stirred for 5 h *via* mechanical agitation of an oil bath at 50 °C. Subsequently, the mixture was diluted fivefold with distilled water. The resulting suspension was centrifuged (centrifuge speed: 10 000 rpm) for 10 min to stop the hydrolysis reaction. The suspension was then dialyzed with distilled water for several days until a pH value of 7.0 was reached, and freeze-dried afterward.^27^ Prior to the reaction process, CNC was dried for 5 h in a vacuum oven at 40 °C for 5 h. Subsequently, 1.0 g CNC and 13 mL DMF (AR) were placed in a three-necked flask attached to a condenser. The mixture was then dispersed *via* constant stirring for 1 h at room temperature in an ultrasonic machine. The three-necked flask was then placed in an oil pot at 100 °C. Chemical modification was performed with a solution containing a fixed amount of acid anhydride, such as acetic anhydride, butyric anhydride, and caproic anhydride (molar fraction of CNC–OH to acid anhydride is 1 : 2). A portion (0.005 g) H_2_SO_4_ (98.08%) acted as the catalyst in the mixture, which was constantly stirred for 4 h. Afterward, the product was cooled to room temperature, and purified *via* washing with acetone solution to eliminate unreacted compounds. Modified-CNC such as acetic-anhydride-modified-CNC, butyric-anhydride-modified-CNC, and caproic-anhydride-modified-CNC, denoted as a-CNC, b-CNC, and c-CNC, respectively, were dehumidified at 45 °C. The modified-CNC was kept for 24 h into a soxhlet extractor at 120 °C with acetone as the extraction agent, and then dried in a vacuum oven at 45 °C.

### Preparation of PBS/modified-CNC composites

Prior to use, the materials were all dried for 48 h in a vacuum oven at 50 °C. The composites were then placed in the double screw extruder (SJZS-10, Wuhan Experiment Instrument Co., Ltd. China), which was operated at a screw rotation speed of 35 rpm. The extrusion temperature of the first district, second district, third district, fourth district, and fifth district were 125 °C, 130 °C, 135 °C, 140 °C, and 146 °C, respectively. After extrusion, the mixture was granulated by a pelleting machine (SHJ-20 model). In accordance with the ASTM Standard D638-03 for Sample Type IV, stretch samples were prepared *via* injection molding (SU125X100 model), performed under the following conditions: furnace-piston temperature: 125 °C, injection pressure: 550 bar, and filling time: 15 s. The PBS composites were prepared from 20.0 g PBS matrix and modified-CNC (component ratios: b-CNC: 0.5, 1.0, 3.0, 5.0, 7.0 wt%; a-CNC: 3.0 wt%; c-CNC: 3.0 wt%).

### Characterization

The particle size distribution of CNC was determined *via* DLS performed at 25 °C.

The morphology of modified-CNC and PBS/modified-CNC composites was observed on *via* scanning electron microscopy (SEM; FEI Quanta 250) performed at an accelerating voltage of 10 kV. Prior to observation, the sample surfaces were sputtered with a layer of gold. The fraction of carbon (C) present in the modified-CNC was determined *via* energy dispersive X-ray analysis (EDAX; Elementar Vario EL Cube, Germany).

Infrared spectra from KBr pellets were obtained on a Fourier Transform Infrared (FT-IR) spectrometer (Nicolet T6670; Thermo Fisher Scientific, the United States) spectra were collected for wavenumbers ranging from 3700 to 500 cm^−1^ at a resolution of 4 cm^−1^.

The room-temperature structure of the modified-CNC was confirmed *via* carbon nuclear magnetic resonance (^13^C NMR) spectroscopy performed on a 400 MHz NMR spectrometer (Bruker AV II-400, Bruker, Germany).

CNC, a-CNC, b-CNC, and c-CNC powder were pressed into round pellets using a tablet press (YP-2 model; Mountains Scientific Instrument Co., Ltd, Shanghai China). The contact angles of water and methylene iodide on each material were measured using a contact angle goniometer (YIKE-360A model; Chengde Precision Test Instrument Factory, China). In addition, the surface free energies of each material were calculated from the Owen–Wendt equation and Young's equation.

Thermogravimetric analysis (TGA) of CNC and the modified-CNC was performed with a thermal analyzer (TA Instruments TGA Q500 V20.13 Build 39). During the measurements, each sample was heated from room temperature to 600 °C at a heating rate of 10 °C min^−1^, under nitrogen atmosphere.

The thermal properties of pure PBS, PBS/a-CNC, PBS/b-CNC, and PBS/c-CNC composites were measured *via* differential scanning calorimetry (DSC, Q2000 V24.11 Build 124) of 3–10 mg samples. The samples were measured at a scanning rate of 10 °C min^−1^ in a sealed aluminum pot under a protective nitrogen atmosphere (calibration: indium standard). The initial sample was held for 5 min at 140 °C to erase the thermal history, and then cooled from 140 to 20 °C as the first cooling run. Subsequently, the sample reheated from 20 to 140 °C during the second heating scan, and the exothermic curves were recorded for analysis.

The X-ray diffraction (XRD) patterns of the PBS/modified-CNC composites were obtained with a DX-1000 diffractometer (Dandong Fangyuan Instrument Co., Ltd, China) using CuKα radiation. The voltage and current were set to 40 kV and 25 mA, respectively. Diffraction patterns were recorded at a scanning rate of 2° min^−1^ for 2*θ* values ranging from 2° to 65°.

The mechanical properties of pure PBS, PBS/a-CNC, PBS/b-CNC, and PBS/c-CNC composites were measured on an Electronic Universal Testing Machine (UTM4204) with large rotational deformation. Tests were performed at room temperature and a tensile speed of 50 mm min^−1^ on dumbbell-shaped samples (gage length between grips: was 25 mm). Five samples were measured for each material, and the average of the five measurement results was reported for all samples.

## Results and discussion

**Fig. 1:**
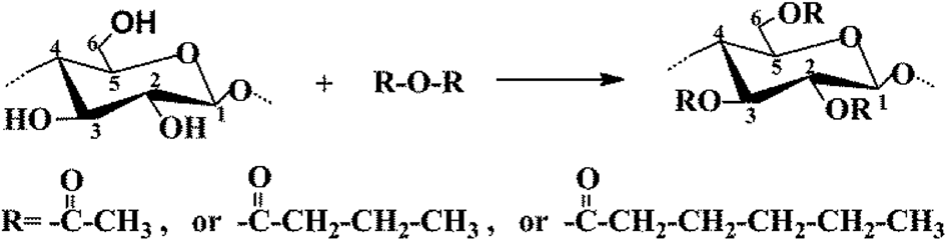
Chemical process of modified CNC.

**Fig. 2:**
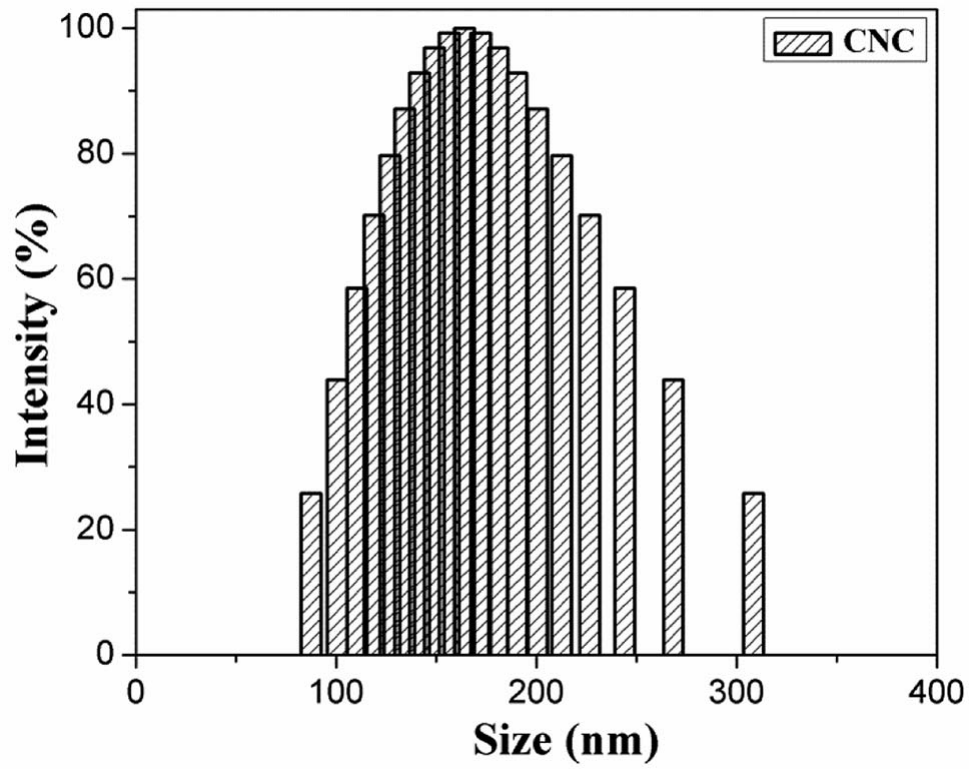
DLS diagrams of CNC.

The CNC surface underwent esterification reactions (see schematic in Fig. 1) with chain anhydrides (such as acetic anhydride, butyric anhydride, and caproic anhydride) of different lengths. The esterification reaction occurs more easily than other reactions. The DLS-determined particle size distribution (range of sizes: 80–130 nm) of CNC is shown in Fig. 2.

### SEM-EDAX analysis

**Fig. 3:**
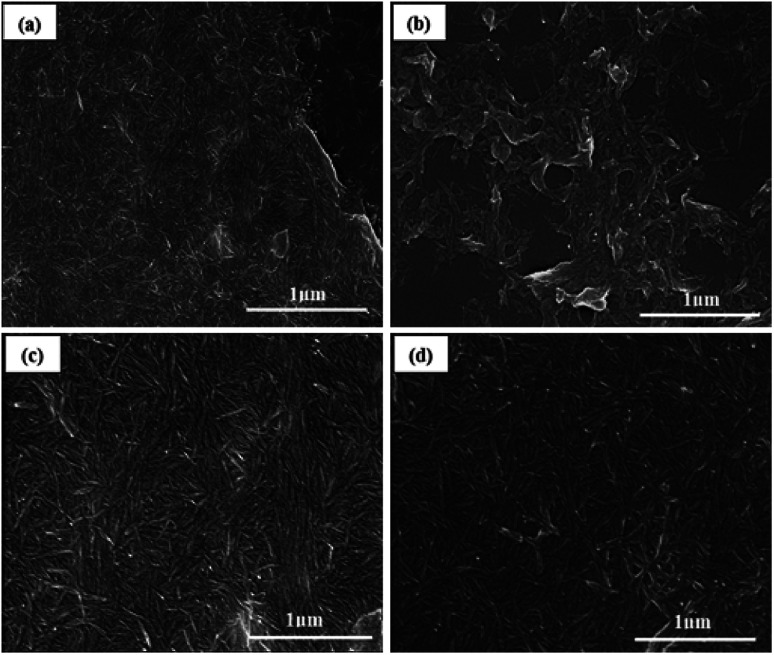
SEM images of (a) CNC, (b) a-CNC, (c) b-CNC, and (d) c-CNC.

Scanning electron microscopy revealed the rod-like morphology of CNC, a-CNC, b-CNC, and c-CNC [see Fig. 3(a–d)]. All three materials consisted of nanometer-sized particle indicating that the acid anhydride modified CNC without destroying the original (CNC) structure.

The influence of acid anhydrides on modified-CNC was investigated by evaluating the C elementary percentage of CNC, a-CNC, b-CNC, and c-CNC (Table 1). During this investigation, the C elementary percentage at three points on each sample was determined *via* SEM-EDAX. As Table 1 shows, an average percentage value of 83.61% was obtained for CNC. This value is lower than the average values obtained for a-CNC (87.09%), b-CNC (88.31%), and c-CNC (89.60%), indicating that CNC surfaces were successfully modified by acetic anhydride, butyric anhydride and caproic anhydride, respectively. Moreover, the C elementary percentage increased with increasing chain length of each acid anhydride.

**Table 1:** C-atomic percentage values of CNC, a-CNC, b-CNC, and c-CNC

C atom	CNC	a-CNC	b-CNC	c-CNC
At%	84.20	88.10	88.10	90.14
	81.28	86.79	86.79	89.59
	85.35	86.38	90.03	89.07
Average at%	83.61	87.09	88.13	89.60

### FT-IR spectral study

**Fig. 4:**
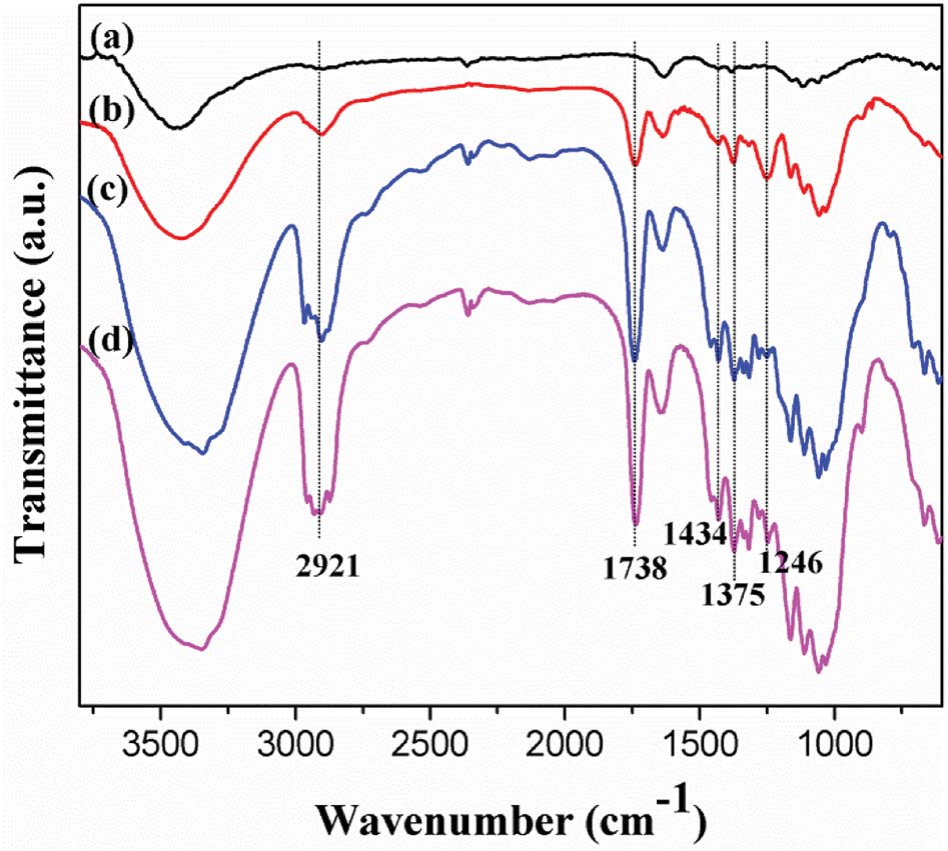
FT-IR analysis of (a) CNC, (b) a-CNC, (c) b-CNC, and (d) c-CNC.

As hydroxyl groups reacted with acid anhydride in the presence of sulfuric acid, the oxygen atoms of CNC underwent acid-induced deprotonation to O^−^ ions, and then reacted with the acid anhydride through an esterification reaction. The characteristic peaks of ester groups, manifested as the sp^3^ C–H stretching vibration at 2921 cm^−1^, were confirmed *via* FT-IR spectroscopy. Similarly, C–H bending vibration peaks corresponding to –CH_3_ in-plane bending and CH_2_ bending occurred at 1375 cm^−1^ and 1434 cm^−1^, respectively, as shown in Fig. 4.^28^

The esterification of CNC was characterized by the emergence of a new peak at 1738 cm^−1^ corresponding to the carbonyl (C=O) associated with the formed ester group. Similarly, the new absorption band at 1246 cm^−1^ was attributed to the C–O stretch vibration of the carbonyl. Lack of changes in the peaks corresponding to CNC, suggests that the esterification reaction was successfully.

### Solid-state NMR analysis

**Fig. 5:**
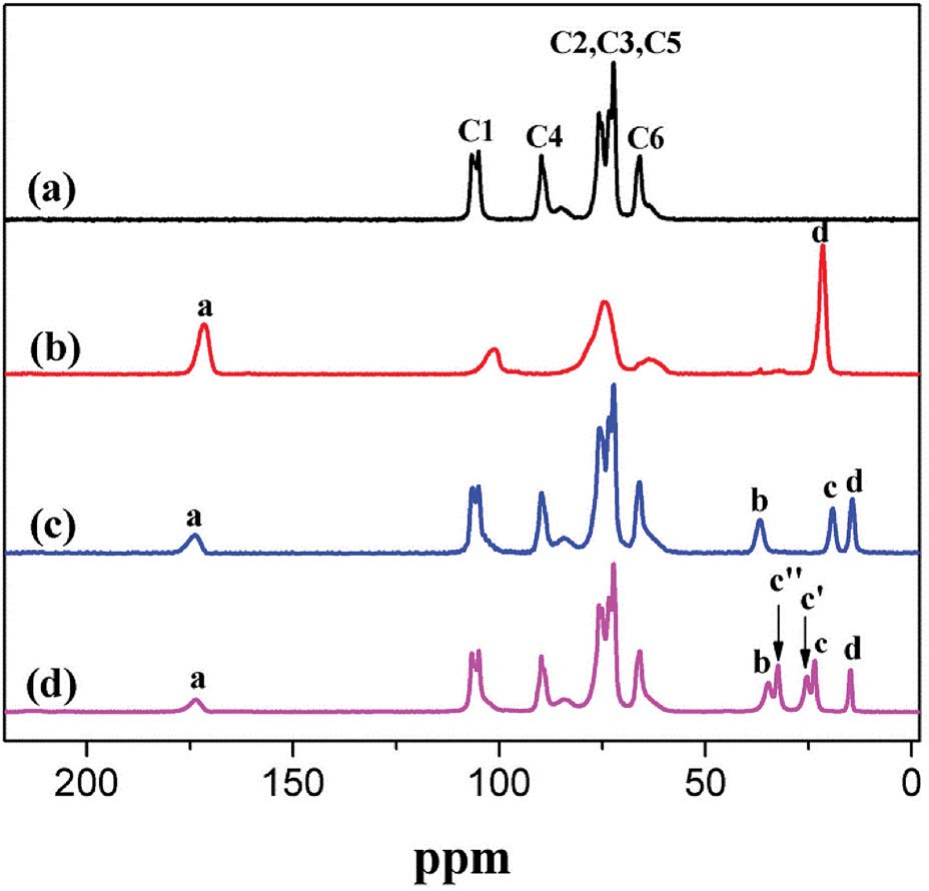
Solid-state ^13^C NMR analysis of (a) CNC, (b) a-CNC, (c) b-CNC, and (d) c-CNC.

The esterification of CNC was also investigated *via*^13^C NMR spectroscopy. The typical ^13^C NMR peaks of CNC were as follows (ppm): 105 (C1), 89 (C4 crystalline), 89 (C4 amorphous), 72 and 75 (C2/C3/C5) as well as 65 (C6 crystalline).^4^Table 2 and Fig. 5 show the peaks associated with the O–C=O, –CH_2_–, and –CH_3_ of a-CNC, b-CNC, and c-CNC. The esterification reaction of CNC was characterized by the emergence of a new peak, at 173 ppm, corresponding to the ester group (O–C=O). The typical ^13^C NMR peaks of –CH_2_– and –CH_3_ associated with all three materials occurred at 21 ppm and 14 ppm, respectively. The esterification of CNC was successfully achieved, as confirmed by the FT-IR and ^13^C NMR data.

**Table 2:** ^13^C NMP chemical shifts and assignment

Substituted ester group	Chemical shift(ppm)	Assignment			Integral value		
		a-CNC	b-CNC	c-CNC	a-CNC	b-CNC	c-CNC
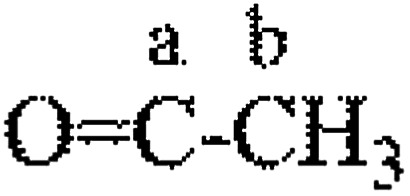	173	a	a	a	1.00	1.00	1.00
	36	—	b	b	—	1.03	1.00
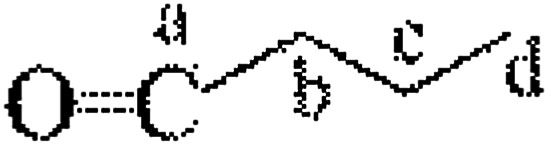	32	—	—	c′′	—	—	1.03
	25	—	—	c′	—	—	0.95
	23	—	—	c	—	—	0.97
	21	b	—	—	57.50	—	—
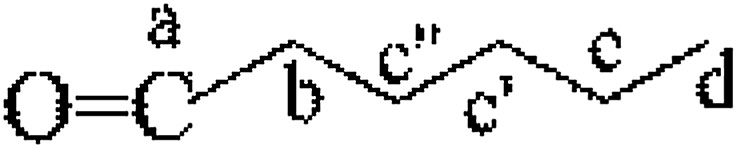	19	—	c	—	—	0.99	—
	14	—	d	d	—	1.05	0.65

### Contact angle measurement and surface free energy of modified-CNC

The polarity and surface energy of modified-CNC were determined *via* contact angle measurements, which allowed comparison of the relative hydrophilicity or hydrophobicity of each sample.

**Fig. 6:**
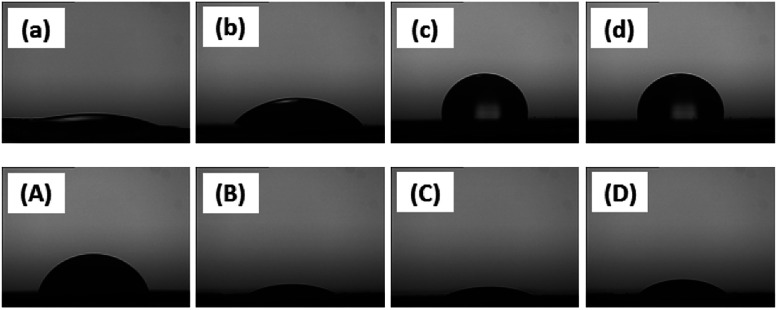
Water contact angle of (a) CNC, (b) a-CNC, (c) b-CNC, (d) c-CNC; methylene iodide contact angle of (A) CNC, (B) a-CNC, (C) b-CNC, (D) c-CNC.

The average contact angles of water and methylene iodide on CNC and modified-CNC are summarized in Table 3. As Fig. 6 shows, the respective contact angles of water and methylene iodide on CNC, a-CNC, b-CNC, and c-CNC after 30 s.

**Table 3:** Contact angle with water and methylene iodide and surface energy

Samples	Water (°)	Methylene iodide (°)	Dispersion force (mJ m^−2^)	Polar force (mJ m^−2^)	Total force (mJ m^−2^)
CNC	≤5	40.26	24.30	47.75	72.05
a-CNC	≤5	28.92	29.16	43.56	72.72
b-CNC	76.20	28.24	41.09	4.28	45.37
c-CNC	73.3	29.60	39.69	5.71	45.40

The water contact angle of CNC and the water contact angle of a-CNC were both lower than 5° after 30 s and 90 s, respectively. However, after 120 s, the water contact angles of b-CNC and c-CNC were 76.2° and 73.3°, respectively, owing to the fact that long ester chain induces hydrophobicity in the hydrophilic CNC. Furthermore, the respective contact angles (28.92°, 28.24°, and 29.6°) of methylene iodide on a-CNC, b-CNC, and c-CNC were lower than that of methylene iodide on CNC (Table 3). Each of these angles decreased to <5° after 180 s. The surface free energies of CNC, a-CNC, b-CNC, and c-CNC were calculated from:^29^

1 Young's equation: *γ*_s_ = *γ*_l_ cos *θ* + *γ*_sl_

2 Dupréus equation: *W* = *γ*_s_ + *γ*_l_ − *γ*_sl_

3 Young–Dupré equation: *W* = *γ*_l_(1 + cos *θ*)

Owen–Wendt equation:

4 *γ*_sl_ = *γ*_s_ + *γ*_l_ − 2(*γ*^d^_s_*γ*^d^_l_)^0.5^ − 2(*γ*^d^_s_*γ*^d^_l_)^0.5^

where, *θ*, *γ*_s_, *γ*_l_, *γ*_sl_, and *W* denote the contact angle of water and methylene iodide on the samples, solid surface free energy, liquid surface free energy, solid–liquid interfacial free energy, and work of adhesion of water on a horizontal surface of the samples, respectively; *γ*^d^_s_ and *γ*^p^_s_ are the dispersive (van der Waals) contribution and the polar (acid–base) contribution, respectively, to the surface energy. Similarly, *γ*^d^_l_ and *γ*^p^_l_ are the dispersive and polar contributions to the liquid, water, and methylene iodide, respectively. Rewriting Young's equation [eqn (1)] in terms of *γ*_sl_ and substituting the resulting expression into the Owen–Wendt equation [eqn (4)] yields: *γ*_l_(1 + cos *θ*) = 2(*γ*^d^_s_*γ*^d^_l_)^0.5^ + 2(*γ*^d^_s_*γ*^d^_l_)^0.5^

Calculations of the surface free energy yielded *γ*_l_, *γ*_ld,_ and *γ*_lp_ values of 72.2, 22.0, and 50.2 mJ m^−2^, respectively, for water and corresponding values of 50.8, 48.5, and 2.30 mJ m^−2^ for methylene iodide.^29^ As Table 3 shows the surface free energy values of b-CNC and c-CNC are considerably lower than those of CNC and a-CNC. This decrease in surface energy contributed significantly to the reduction of the polar component, indicating that (i) the hydroxyl groups were substituted with non-polar ester groups and (ii) b-CNC and c-CNC were well dispersed in the non-polar polymer.

### Thermal behaviors and stability

**Fig. 7:**
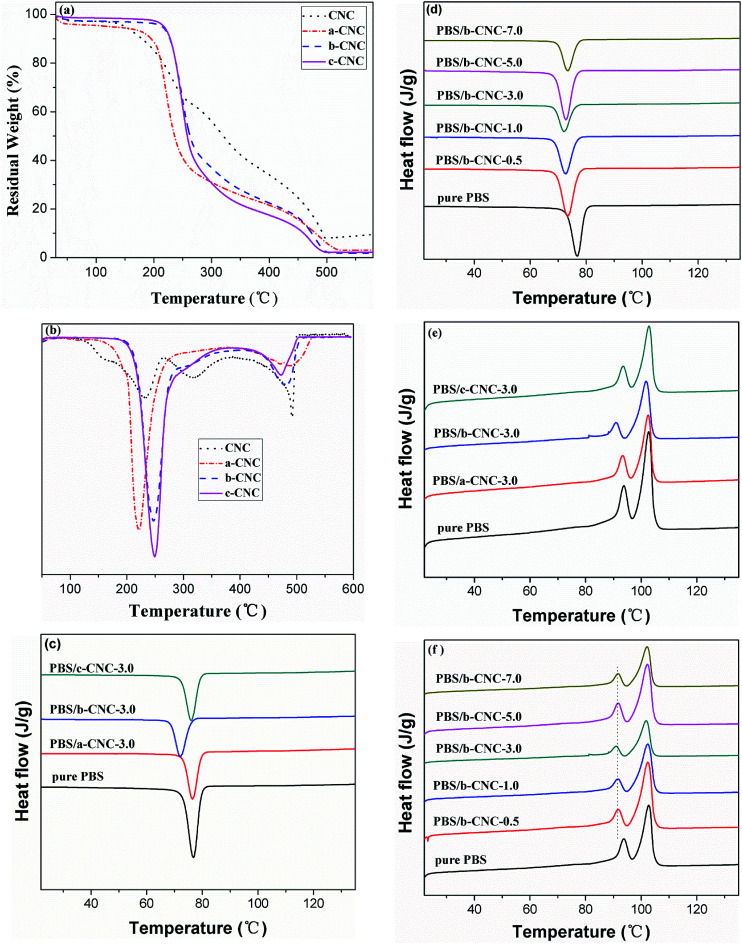
(a) TGA and (b) DTG curves of CNC, a-CNC, b-CNC, and c-CNC; (c and d) DSC cooling and (e and f) the second heating scans of pure PBS and PBS/modified CNC composites.

The thermal stability of CNC and modified-CNC in nitrogen atmosphere was determined *via* TGA. The TGA thermogram of each sample is shown in Fig. 7(a), and the relevant data obtained from the TGA curves are summarized in Table 4. The CNC, a-CNC, b-CNC, and c-CNC samples all exhibited the same decomposition outline, indicating that the decomposition process of modified-CNC was defined primarily by the CNC substrate. As Table 4 shows, the decomposition temperature of a-CNC is lower than that of CNC. However the initial degradation temperature and maximum degradation temperature of CNC are lower and higher, respectively, than those of b-CNC and c-CNC. The thermal stabilities of b-CNC and c-CNC at the initial degradation temperatures were superior to that of CNC. Those results from the fact that the initial decomposition temperature based on 5 wt% mass loss (*T*_5%_) of b-CNC and c-CNC is higher than that of the CNC substrate. For example, degradation of pure CNC began at 149.93 °C, which is significantly lower than the *T*_5%_ values (213.85 °C and 217.26 °C) of b-CNC and c-CNC, respectively. The b-CNC and c-CNC materials can therefore be processed and used at higher temperatures (than the pure CNC) before initial thermal degradation begins. In addition, the 50 wt% mass loss temperature (*T*_50%_) values of all three modified materials were lower than that of the CNC substrate. Degradation of CNC began at 320.53 °C, which is considerably higher than the *T*_50%_ values (235.94 °C, 262.70 °C, and 257.95 °C) of a-CNC, b-CNC, and c-CNC, respectively. The modified-CNC samples may have decomposed at lower temperatures than the pure CNC, owing to the fact that the surface became porous (due to the substituted ester chains). The interior of the CNC was then easily penetrated (owing to this looseness). The results indicated that b-CNC and c-CNC exhibited considerably higher thermal stability than CNC at the initial decomposition temperature. Among the *T*_50%_ values of the modified materials, the best value was obtained for b-CNC.

**Table 4:** Thermogravimetric data of CNC, a-CNC, b-CNC, and c-CNC in nitrogen

Samples	CNC	a-CNC	b-CNC	c-CNC
*T*_onset_ a (°C)	149.93	121.99	213.85	217.26
*T*_50%_ (°C)	320.53	235.94	162.70	257.95
Residues at 590 °C (%)	9.81	3.19	1.79	2.24
*T*_max1_	227.97	221.71	246.93	249.17
*T*_max2_	317.87	489.25	484.38	471.54
*T*_max3_	491.89	—	—	—

a *T*_onset_: the initial temperature at 5 wt% mass loss.

The differential thermogravimetric (DTG) curves for CNC, a-CNC, b-CNC, and c-CNC and the decomposition temperatures based on these curves are shown in Fig. 7(b) and listed in Table 4, respectively. As the Fig. 7(b) shows, CNC exhibited greater thermal stability than a-CNC at *T*_max1_, but less stability than b-CNC and c-CNC. In contrast to CNC, the modified materials decompose at two ranges of temperatures, characterized by maximum temperatures of *T*_max1_ and *T*_max2_. Residues of 3.19, 1.79, and 2.24% were obtained for a-CNC, b-CNC and c-CNC, respectively. The CNC was characterized by three maximal decomposition temperatures (referred to as *T*_max1_, *T*_max2_, and *T*_max3_). This resulted possibly from the broad particle size distribution and molecular weight distribution of CNC *i.e.*, the decomposition temperature would vary with the molecular weight. Table 4 reveals *T*_max1_ values of 227.97 °C, 221.71 °C, 246.93 °C, and 249.17 °C, as well as *T*_max2_ values of 317.87 °C, 489.25 °C, 484.38 °C, and 471.54 °C for CNC, a-CNC, b-CNC and c-CNC, respectively. These *T*_max1_ values indicate that the thermal stability of b-CNC and c-CNC is greater than that of CNC while, in the case of *T*_max2_, the thermal stability of all three modified materials is greater than that of CNC. At *T*_max3_ (491.89 °C), decomposition occurred only in the case of CNC. In fact, at decomposition temperatures lower than *T*_max3,_ the thermal stability of CNC was lower than that of b-CNC and c-CNC. This resulted from the fact that hydrogen bonds in CNC were partially substituted by ester groups. Therefore, for graft acid anhydride, the thermal stability fluctuated with increasing length of the esterification chains, with b-CNC exhibiting better thermal stability than a-CNC and c-CNC.

Improved interfacial adhesion between the polymer matrix and nanoparticles would have a significant influence on the thermal performance and crystallization behavior of the matrix. Therefore, DSC-based investigation focused on determining the effect of a-CNC, b-CNC, and c-CNC content on the thermal performance of PBS was warranted. The DSC cooling curves of pure PBS, PBS/a-CNC, PBS/b-CNC, and PBS/c-CNC nanocomposites are shown in Fig. 7(c and d). PBS is a typical semi-crystalline polymer. The non-isothermal crystallization of pure PBS, PBS/a-CNC, and PBS/c-CNC composites is characterized by an exothermic peak that occurs at the crystallization peak temperature (*T*_c_, in this case, 69.8 °C). The *T*_c_ of pure PBS, PBS/a-CNC, and PBS/c-CNC composites varied only modestly, indicating that the crystallization rate of PBS remained approximately constant with a-CNC and c-CNC nanoparticle filling. However, comparing with the *T*_c_ resulting from a-CNC and c-CNC filling, the *T*_c_ shifted by larger amounts after the incorporation of b-CNC nanoparticles. With the addition of 3.0 wt% b-CNC nanoparticles, the *T*_c_ of PBS decreased to 67.1 °C. This change indicated that crystallization depends possibly on the mobility of PBS molecular chains in the composites. These chains were possibly confined by the greater number of ester groups in b-CNC nanoparticles (compared with the number in a-CNC and c-CNC nanoparticles). As Fig. 7(d) shows when the b-CNC content is increased, the *T*_c_ of PBS/b-CNC composites decreased slightly to lower temperatures than the *T*_c_ of pure PBS. This decrease may be attributed to the excellent dispersion of b-CNC nanoparticles in PBS and, in turn, possible increase in the viscosity. However, the mobility of the PBS molecular chain decreased leading to a strong interaction between the well-dispersed b-CNC nanoparticles and the PBS matrix. This interaction, in turn, hindered crystallization. The DSC traces obtained in the second heating run of pure PBS and PBS/modified-CNC composites are shown in Fig. 7(e and f). The Fig. 7(e) reveals double melting peaks and small variations in the melting temperature (*T*_m_) of pure PBS, PBS/a-CNC, and PBS/c-CNC composites. The minor peak (*T*_m1_) and the major peak (*T*_m2_) occur at 93.3 °C and 102.6 °C, respectively. However, the *T*_m_ of PBS/b-CNC composites decreased slightly with increasing b-CNC content to values lower than the *T*_m_ of pure PBS. The *T*_m1_ and *T*_m2_ of the PBS/b-CNC composites occurred at 90.8 °C and 101.5 °C, respectively. As Fig. 7(f) shows, the peak temperature of *T*_m1_ shifted gradually to lower values with increasing content of b-CNC nanoparticles, whereas *T*_m2_ remained constant at 101.5 °C. The double melting peaks may have resulted from the melting-recrystallization mechanism. The *T*_m1_ peak resulted from the melting of initial crystals formed prior to the second DSC scan, and the *T*_m2_ peak resulted from the melting of recrystallized crystals during the heating scan. In other words, the heating process consisted of competing melting and recrystallization processes. This could be explained by the fact that unstable crystals melted first and more stable crystals melted at higher temperatures than the melting temperatures of these unstable crystals. Hence, the melting rate was faster than the crystallization rate, and the crystallization rate decreased at high temperatures, resulting in an endothermic peak.

### The wide-angle XRD analysis

**Fig. 8:**
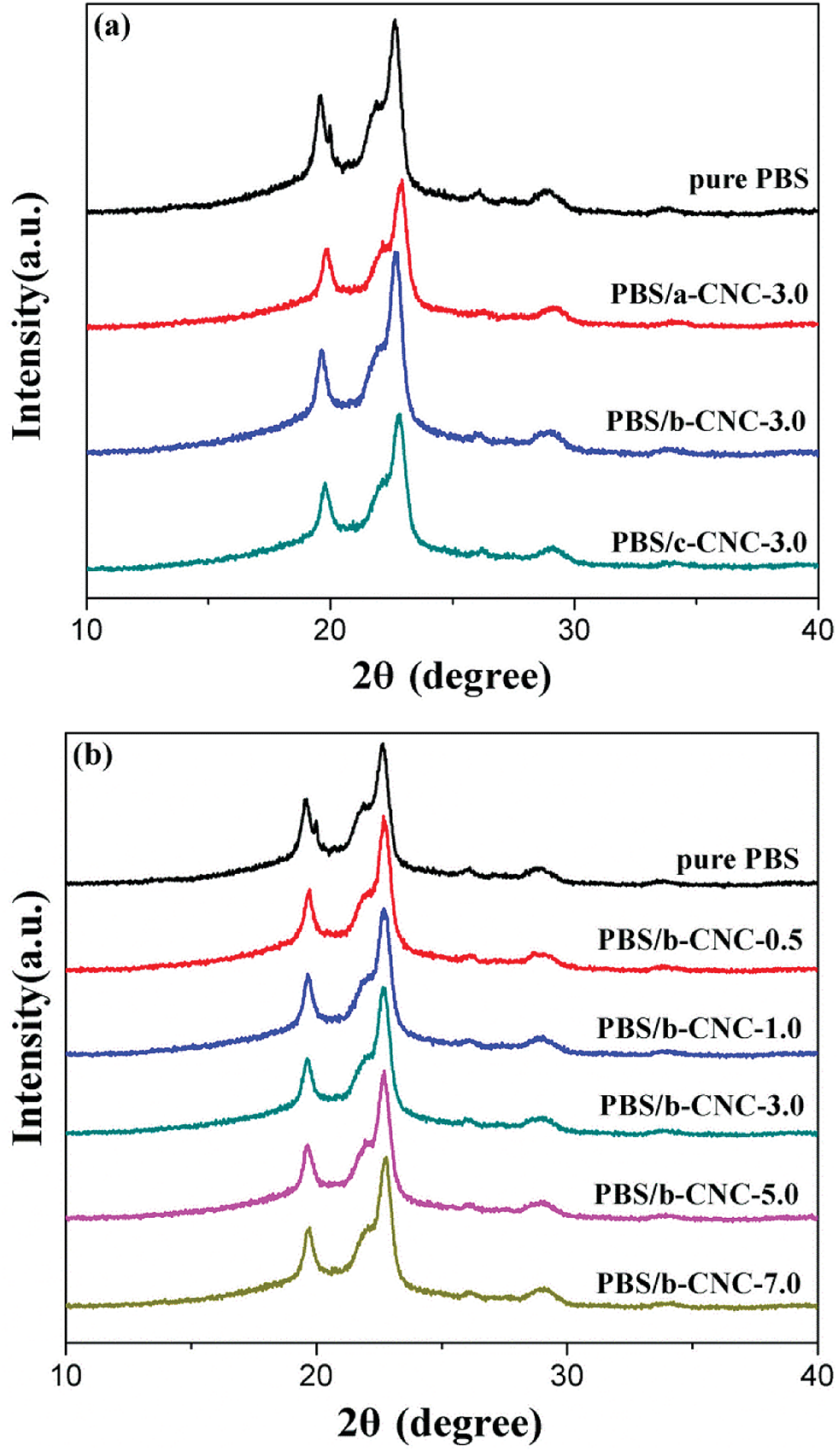
XRD patterns of (a) pure PBS, PBS/a-CNC-3.0, PBS/b-CNC-3.0, and PBS/c-CNC-3.0 composites and (b) PBS/b-CNC composites with different b-CNC contents.

The XRD spectra of pure PBS and PBS/modified-CNC composites are shown in Fig. 8(a and b). Pure PBS exhibited three main characteristic diffraction peaks at 2*θ* of 19.7, 21.9, and 22.6° corresponding to the (020), (021), and (110) planes of PBS, respectively.^30,31^ The locations of these peaks were maintained with incorporation of modified-CNC nanoparticles. In other words, the crystal structure of PBS was retained with the addition of modified-CNC, regardless of the b-CNC contents.

### Dispersion of modified-CNC in the PBS matrix

**Fig. 9:**
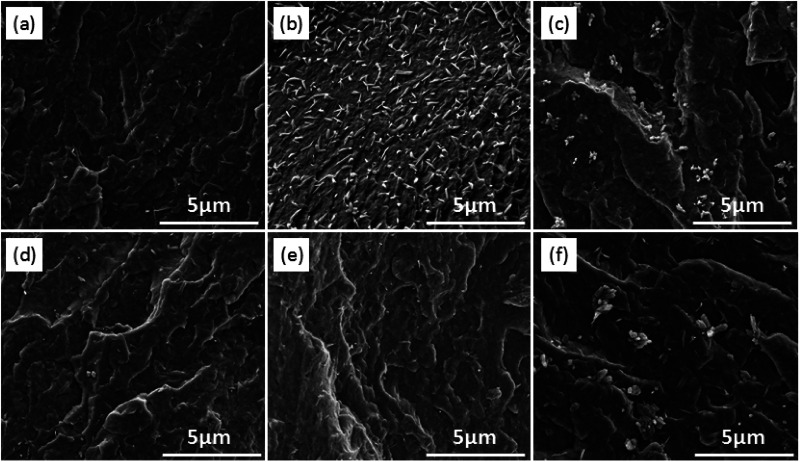
SEM images of (a) PBS/a-CNC-3.0, (b) PBS/b-CNC-3.0, (c) PBS/c-CNC-3.0, (d) PBS/b-CNC-0.5, (e) PBS/b-CNC-1.0, (f) PBS/b-CNC-5.0.

Scanning electron micrographs showing the cryo-fractured surfaces of the PBS/modified-CNC composites are shown in Fig. 9. The pure PBS and the PBS/modified-CNC composites are characterized by a glossy surface and rough surfaces, respectively, as shown in Fig. 9(a–f). From Fig. 9(a–c), b-CNC nanoparticles were dispersed very well in PBS matrix in contrast to a-CNC and c-CNC nanoparticles. The a-CNC and c-CNC nanoparticles are partly dispersed and poorly dispersed, respectively, in the matrix. This poor dispersion may have resulted from the high viscosity of the c-CNC with long ester chain segments. In addition, the roughness increased with increasing b-CNC loading, and the particle dispersion was maintained (aggregation was absent) on the surface even for b-CNC contents of up to 3.0 wt% [Fig. 9(b, d and e)]. Strong interfacial adhesion occurred between the well-dispersed b-CNC nanoparticles and the PBS matrix. However, moderate aggregation occurred on the surface for b-CNC loadings increased to 5.0 wt%, as shown in Fig. 9(f), thereby confirming that the optimum b-CNC content is 3 wt%.

### Mechanical properties

**Fig. 10:**
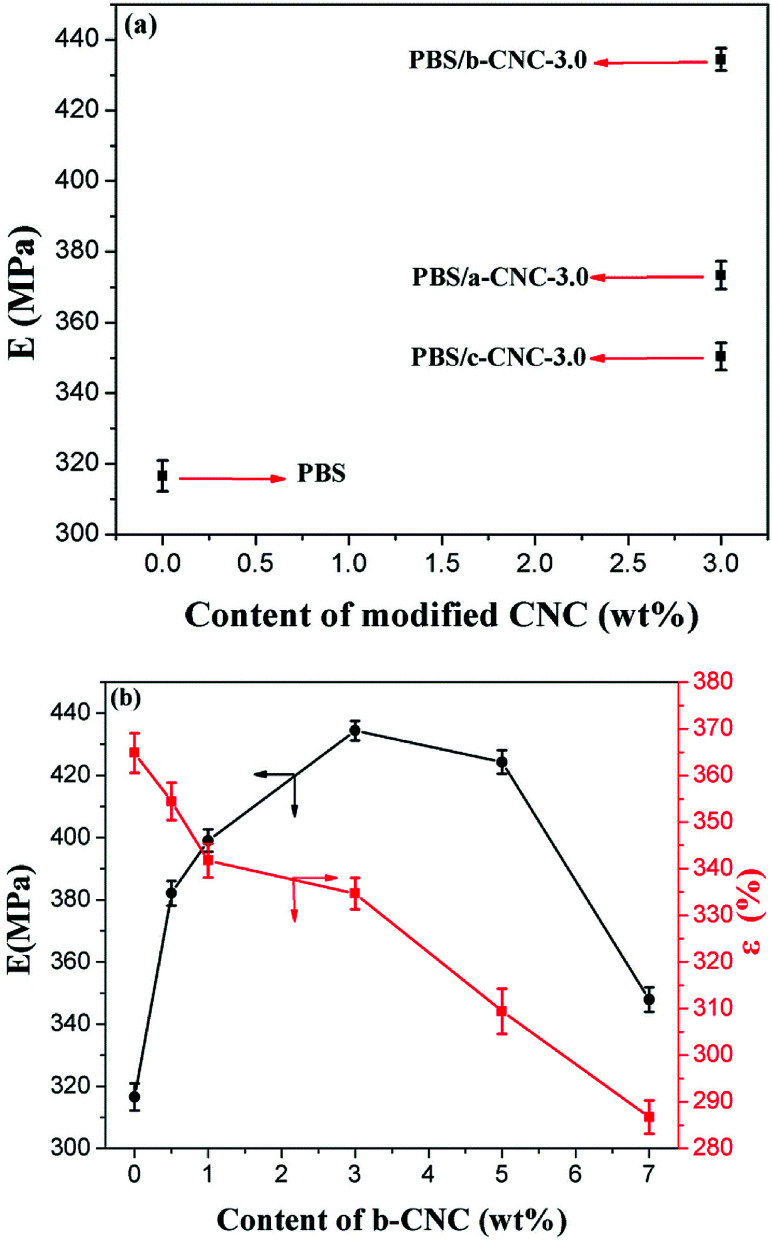
Effect of (a) the a-CNC, b-CNC, and c-CNC content on the tensile modulus of the PBS composites and (b) b-CNC content on the tensile modulus and elongation at fracture of the PBS/b-CNC composites.

PBS has high toughness, but low tensile strength. The effect of a-CNC, b-CNC, and c-CNC on PBS was investigated *via* tensile testing. As Fig. 10 shows, the effect of (a) a-CNC, b-CNC, and c-CNC content on the tensile modulus (*E*) of PBS composites and (b) b-CNC content on the *E* and the elongation at fracture (*ε*) of PBS/b-CNC composites.

As Fig. 10(a) shows, *E* values of 316.5 ± 4.3, 373.3 ± 3.9, 434 ± 3.1, and 350 ± 3.8 MPa are obtained for PBS, PBS/a-CNC-3.0, PBS/b-CNC-3.0, and PBS/c-CNC-3.0, respectively. These values indicate that, consistent with an enhancement of the mechanical properties, the *E* of the PBS mixed with modified- CNC is higher than that of pure PBS.

The highest *E* was obtained for the PBS/b-CNC composite. This indicated that b-CNC and PBS were better mixed (than either a-CNC or c-CNC with PBS) and the dispersion of b-CNC in PBS was the best. The three-dimensional network structure was formed by mixing modified-CNC with PBS, thereby increasing the effect of b-CNC nanoparticles (added in only low volume fractions) on the mechanical properties of PBS. Correspondingly, the *E* of PBS/b-CNC composites was higher than that of pure PBS [see inset of Fig. 10(b)]. *E* values of 382.1 ± 4.0, 399.0 ± 11, 434 ± 3.1, 424.3 ± 3.7, and 347 ± 3.9 MPa were obtained for PBS/b-CNC-0.5, PBS/b-CNC-1.0, PBS/b-CNC-3.0 PBS/b-CNC-5.0, and PBS/CNC-7.0, respectively. The optimum mass fraction of b-CNC nanoparticles was found to be 3.0 wt%. In other words, b-CNC additions exceeding 3.0 wt% resulted in agglomeration of the nanoparticles, leading to poor interface compatibility of PBS with these particles and, in turn, a decrease in the tensile modulus. Moreover, the *ε* decreased gradually with increasing b-CNC content. For example, *ε* values of 364.8 ± 4.2, 354.4 ± 4.0, 341.7 ± 3.6, 334.6 ± 3.3, 309 ± 4.8, and 286 ± 3.5% were obtained for PBS, PBS/b-CNC-0.5, PBS/b-CNC-1.0, PBS/b-CNC-3.0, PBS/b-CNC-5.0, and PBS/b-CNC-7.0, respectively. The *E* value of PBS/b-CNC composites was higher than that of pure PBS (consistent with enhancement of the mechanical properties) and increased with increasing b-CNC contents. The *E* value of pure PBS (*i.e.*, 316 ± 15 MPa) improved by up to 26.7% with the addition of 3.0 wt% b-CNC. Therefore, the tensile modulus of PBS was significantly enhanced, owing to the incorporation of modified-CNC, whereas the elongation at fracture decreased considerably.

## Conclusions

Modified-CNC materials were successfully prepared by means of an esterification reaction with acetic anhydride, butyric anhydride, and caproic anhydride, respectively. The rod-like morphology of these materials, which consisted of nanometer-sized particles, was confirmed *via* SEM. Furthermore, EDAX, FT-IR, and ^13^C NMR analyses confirmed that the esterification reaction was successfully achieved *i.e.*, the hydroxyls were substituted by ester groups. Contact angle and surface free energy measurements of a-CNC, b-CNC, and c-CNC nanoparticles revealed that the relative hydrophilicity of the modified-CNC materials was significantly lower than that of CNC. The *T*_5%_ values (213.85 °C and 217.26 °C, respectively) of the b-CNC and c-CNC composites were both higher than that of the pure CNC. PBS matrix with well-dispersed a-CNC, b-CNC, and c-CNC nanoparticles was successfully fabricated *via* melt blending. The addition of a-CNC, b-CNC, and c-CNC nanoparticles had no effect on the isothermal crystallization mechanism and the crystal structure of PBS, as evidenced by the XRD results. In contrast to CNC, a-CNC, and c-CNC nanoparticles, b-CNC was well-dispersed in the PBS matrix, and an optimal fraction of 3.0 wt% was identified. The tensile modulus of PBS increased significantly, owing to incorporation of a-CNC, (especially) b-CNC and c-CNC. However, the elongation at fracture of the PBS/a-CNC, PBS/b-CNC, and PBS/c-CNC composites decreased considerably. In summary, the PBS/b-CNC-3.0 composite exhibited the optimal overall properties (including dispersity and balanced mechanical strength). Therefore, a study of other properties (for example, the hardness, impact strength, and rheological behavior) would be carried out.

## Conflicts of interest

There are no conflicts to declare.

## Supplementary Material
